# Construction and validation of a nomogram model for predicting diabetic peripheral neuropathy

**DOI:** 10.3389/fendo.2024.1419115

**Published:** 2024-12-16

**Authors:** Hanying Liu, Qiao Liu, Mengdie Chen, Chaoyin Lu, Ping Feng

**Affiliations:** Department of Endocrinology, Taizhou Central Hospital (Taizhou University Hospital), Taizhou, China

**Keywords:** diabetic peripheral neuropathy, nomogram, age, hip circumference, fasting plasma glucose, C-peptide, albumin, blood urea nitrogen

## Abstract

**Objective:**

Diabetic peripheral neuropathy (DPN) is a chronic complication of diabetes that can potentially escalate into ulceration, amputation and other severe consequences. The aim of this study was to construct and validate a predictive nomogram model for assessing the risk of DPN development among diabetic patients, thereby facilitating the early identification of high-risk DPN individuals and mitigating the incidence of severe outcomes.

**Methods:**

1185 patients were included in this study from June 2020 to June 2023. All patients underwent peripheral nerve function assessments, of which 801 were diagnosed with DPN. Patients were randomly divided into a training set (n =711) and a validation set (n = 474) with a ratio of 6:4. The least absolute shrinkage and selection operator (LASSO) logistic regression analysis was performed to identify independent risk factors and develop a simple nomogram. Subsequently, the discrimination and clinical value of the nomogram was extensively validated using receiver operating characteristic (ROC) curves, calibration curves and clinical decision curve analyses (DCA).

**Results:**

Following LASSO regression analysis, a nomogram model for predicting the risk of DPN was eventually established based on 7 factors: age (OR = 1.02, 95%CI: 1.01 - 1.03), hip circumference (HC, OR = 0.94, 95%CI: 0.92 – 0.97), fasting plasma glucose (FPG, OR = 1.06, 95%CI: 1.01 - 1.11), fasting C-peptide (FCP, OR = 0.66, 95%CI: 0.56 - 0.77), 2 hour postprandial C-peptide (PCP, OR = 0.78, 95%CI: 0.72 – 0.84), albumin (ALB, OR = 0.90, 95%CI: 0.87 – 0.94) and blood urea nitrogen (BUN, OR = 1.08, 95%CI: 1.01 - 1.17). The areas under the curves (AUC) of the nomogram were 0.703 (95% CI 0.664-0.743) and 0.704 (95% CI 0.652-0.756) in the training and validation sets, respectively. The Hosmer–Lemeshow test and calibration curves revealed high consistency between the predicted and actual results of the nomogram. DCA demonstrated that the nomogram was valuable in clinical practice.

**Conclusions:**

The DPN nomogram prediction model, containing 7 significant variables, has exhibited excellent performance. Its generalization to clinical practice could potentially help in the early detection and prompt intervention for high-risk DPN patients.

## 1 Introduction

Diabetes mellitus (DM) is recognized as a global public health disease, with an extremely high prevalence in both developed and developing countries. Diabetic peripheral neuropathy (DPN) is one of the main chronic complications of DM and significantly affects an individual’s quality of life (1, 2). Patients with DPN can yield serious health ramifications such as numbness or pain in the distal extremities, possibly progressing to diabetic foot or even necessitating amputation (1–6). Therefore, early detection and timely intervention in cases of DPN are crucial in reducing the incidence of adverse outcomes.

Numerous measurements are currently accessible for clinical use in facilitating the diagnosis of DPN; however, bedside foot screening assessment fail to detect subtle neuropathy, scored clinical sensory tests are plagued by a lack of objectivity and standardization, and the gold standard neurophysiological examination is hindered by its high cost and time consumption (1–8). Consequently, the application of these methods is limited in wide-scale screening activities or routine clinical practice. It is therefore imperative to develop an accessible, objective and accurate tool to facilitate the early detection and quick assessment of DPN risk in restricted clinical settings.

Thus, our goal was to construct a straightforward and visual nomogram model utilizing assorted clinical variables to predict the risk of developing DPN. Such a model would empower clinicians in identifying high-risk individuals early and providing effective treatments in time to enhance clinical prognosis.

## 2 Subjects and methods

### 2.1 Subjects

In this cross-sectional study, a total of 1185 individuals aged 18-75 years were recruited from June 2020 to June 2023 at Taizhou Central Hospital (Taizhou University Hospital), China. The subjects included were diagnosed with diabetes according to the World Health Organization guidelines of 1999 and all participants were capable of independent communication. Patients with a history of drug or medication abuse, acute infectious diseases, malignancies, severe life-threatening illnesses, and other causes contributing to peripheral neuropathy were excluded. The study protocol was approved by the Ethics Committees of Taizhou Central Hospital and conformed to the Helsinki Declaration. Informed consents were obtained from all participants.

### 2.2 Anthropometric and laboratory assessments

A digital sphygmomanometer was used to measure the diastolic blood pressure (DBP) and systolic blood pressure (SBP). The blood pressure was measured at least twice consecutively and the average of the two readings was taken as the final recorded value. The weight and height of participants were measured by a digital scale, and body mass index (BMI) was subsequently calculated as weight (kg) divided by height squared (m^2^). The well-trained examiner performed circumference measurements with the tape placed horizontally and tight to the skin without compressing the soft tissues. Neck circumference (NC) was measured horizontally at the level of the lower border of the laryngeal prominence with the head erect and eyes facing forward. Waist circumference (WC) was measured on the midline between the lowest rib margin and the upper margin of the iliac crest. Hip circumference (HC) was measured at the level of the pubic symphysis to the most convex part of the gluteus maximus muscle.

In addition, fasting pre-elbow venous blood specimens were taken early in the morning after 8 hours of fasting, and patients were requested to eat a low-fat diet and to avoid alcoholic consumption and staying up late the day before blood collection. Hematological and common biochemical examinations of all patients were performed in the same laboratory following standard laboratory methods, including: alanine aminotransferase (ALT), aspartate aminotransferase (AST), alkaline phosphatase (ALP), γ-glutamyl transpeptidase (γ-GT), albumin (ALB), blood urea nitrogen (BUN), serum creatinine (Scr), serum uric acid (SUA), triglyceride (TG), total cholesterol (TC), high-density lipoprotein cholesterol (HDL-c), low-density lipoprotein cholesterol (LDL-c), serum fasting plasma glucose (FPG), 2 hour postprandial plasma glucose (PPG), fasting C-peptide (FCP), 2 hour postprandial C-peptide (PCP), glycated hemoglobin A1c (HbA1c), and hemoglobin (Hb).

### 2.3 Assessment of peripheral nerve function

Nerve conduction studies stand as the gold standard for assessing peripheral nerve function and diagnosing DPN due to the accuracy, reliability, and sensitivity (2–8). Motor and sensory nerve conduction velocities and nerve action potential amplifications of the bilateral median, ulnar, tibial, and common peroneal nerves were measured by experienced technicians. Subsequently, DPN is diagnosed on the basis of the functional abnormalities detected within the aforementioned peripheral nerves (9).

### 2.4 Statistical analysis

The Quantile-Quantile plot (Q–Q plot) was used to determine the normality of the data. Normally distributed continuous variables were expressed as mean ± standard deviation (SD) and non-normally distributed continuous variables were described as median (interquartile range, IQR). Continuous variables were compared using the Student’s t-test or the Mann-Whitney U-test. Categorical variables were expressed as percentages and compared using the Chi-squared or Fisher’s exact test. The least absolute shrinkage and selection operator (LASSO) logistic regression was used for multivariate analysis to reduce the effect of multicollinearity between independent variables. Significant independent risk factors screened by LASSO regression were subsequently entered into multivariate logistic regression to construct a nomogram model. Further, the discriminatory capacity of the nomogram model was assessed by the area under the curve (AUC) of receiver operating characteristic (ROC). The calibration performance was assessed by the calibration curve and the Hosmer-Lemeshow test. The decision curve analysis (DCA) was incorporated to verify the clinical validity of the model. Statistical analyses were performed using SPSS version 26.0 (IBM, Chicago, IL, USA) and R software (version 4.3.2; https://www.R-project.org). P-value < 0.05 in two-tailed test was considered statistically significant.

## 3 Results

### 3.1 Baseline characteristics

In the present study, a comprehensive assessment was undertaken in involving 1185 diabetic patients who fulfilled the inclusion criteria. Of these participants, 801 were diagnosed with DPN, whereas the remaining 384 did not exhibit peripheral neuropathy. All patients were randomly divided into the training set (n = 711) and the validation set (n = 474) at a ratio of 6:4. When developing prediction models for binary outcomes, an established rule of thumb for the required sample size is to ensure at least 10 events for each predictor parameter being considered for inclusion in the prediction model equation. This is widely referred to as needing at least 10 events per variable (10-EPV) (10). There were a total of 27 independent variables in our study; therefore, according to the 10-EPV rule of thumb, the number of positive event cases (i.e., DPN) in the training set for this study should be at least greater than 270. As shown in **Table 1**, there were 467 DPN patients in the final training set of this study, indicating that the study had adequate power to detect significant effects. Upon comparison, the basic characteristics of the two sets revealed no statistically significant differences (p>0.05, **Table 1**). The overall similarity of the cohorts provided a solid foundation for further investigations into predictive modeling and outcomes.

**Table 1:** Comparison of basic characteristics between patients in training and validation sets.

Characteristics	training set (n = 711)	validation set (n = 474)	P-value
Gender (%)			0.645
Male	486 (68.4%)	330 (69.6%)	
Female	225 (31.6%)	144 (30.4%)	
DPN (%)			0.085
No	244 (34.32%)	140 (29.54%)	
Yes	467 (65.68%)	334 (70.46%)	
Age (years)	55.0 (46.0, 63.0)	55.0 (46.0, 63.0)	0.955
DBP (mmHg)	75.0 (72.0, 83.0)	76.0 (74.0, 84.0)	0.195
SBP (mmHg)	126.0 (121.0, 136.0)	126.0 (122.0, 136.0)	0.249
HR	73.0 (70.0, 79.0)	72.0 (70.0, 78.0)	0.537
BMI (kg/m^2^)	24.3 (22.3, 26.7)	24.6 (22.5, 27.0)	0.191
NC (cm)	36.8 ± 3.5	37.0 ± 3.6	0.356
WC (cm)	89.0 ± 10.0	89.0 ± 10.0	0.766
HC (cm)	93.0 (89.0, 98.0)	93.0 (89.0, 97.0)	0.456
FPG (mmol/L)	8.6 (6.4, 11.4)	8.5 (6.7, 11.2)	0.916
PPG (mmol/L)	13.5 ± 4.7	13.6 ± 5.0	0.608
FCP (ng/mL)	1.40 (0.81, 2.02)	1.35 (0.88, 1.94)	0.962
PCP (ng/mL)	2.35 (1.33, 3.82)	2.21 (1.27, 3.85)	0.502
HbA1c (%)	10.30 (8.55, 12.30)	10.50 (8.50, 12.20)	0.873
Hb (g/L)	139.0 ± 19.0	139.0 ± 19.0	0.923
ALT (U/L)	21.0 (15.0, 36.0)	20.0 (14.0, 32.0)	0.060
AST (U/L)	19.0 (15.0, 26.0)	19.0 (15.0, 25.0)	0.903
ALP (U/L)	76.0 (63.0, 92.0)	75.0 (62.0, 90.0)	0.152
γ-GT (U/L)	28.0 (18.0, 44.0)	26.0 (18.0, 41.0)	0.336
ALB (g/L)	40.6 (38.0, 43.7)	40.5 (37.6, 43.0)	0.223
BUN (mmol/L)	5.1 (4.0, 6.5)	5.1 (4.0, 6.1)	0.185
Scr (μmol/L)	65.0 (55.0, 79.0)	66.0 (55.0, 78.0)	0.862
SUA (μmol/L)	314.0 (255.0, 383.0)	313.0 (250.0, 382.0)	0.444
TG (mmol/L)	1.67 (1.15, 2.44)	1.67 (1.11, 2.52)	0.964
TC (mmol/L)	4.60 (3.82, 5.43)	4.58 (3.82, 5.35)	0.573
HDL-c (mmol/L)	0.96 (0.79, 1.16)	0.94 (0.78, 1.13)	0.507
LDL-c (mmol/L)	2.68 (2.05, 3.35)	2.66 (2.09, 3.33)	0.859

Data are expressed as the mean ± SD or the median (IQR); categorical variables are expressed as n, (%).

DPN, diabetic peripheral neuropathy; DBP, diastolic blood pressure; SBP, systolic blood pressure; HR, heart rate; BMI, body mass index; NC, neck circumference; WC, waist circumference; HC, hip circumference; FPG, fasting plasma glucose; PPG, 2 hour postprandial plasma glucose; FCP, fasting C-peptide; PCP, 2 hour postprandial C-peptide; HbA1c, glycated hemoglobin A1c; Hb, hemoglobin; ALT, alanine aminotransferase; AST, aspartate aminotransferase; ALP, alkaline phosphatase; γ-GT, γ-glutamyl transpeptidase; ALB, Albumin; BUN, blood urea nitrogen; Scr, serum creatinine; SUA, serum uric acid; TG, triglyceride; TC, total cholesterol; HDL-c, high-density lipoprotein cholesterol; LDL-c, low-density lipoprotein cholesterol.

### 3.2 LASSO regression analysis

**Figure 1:**
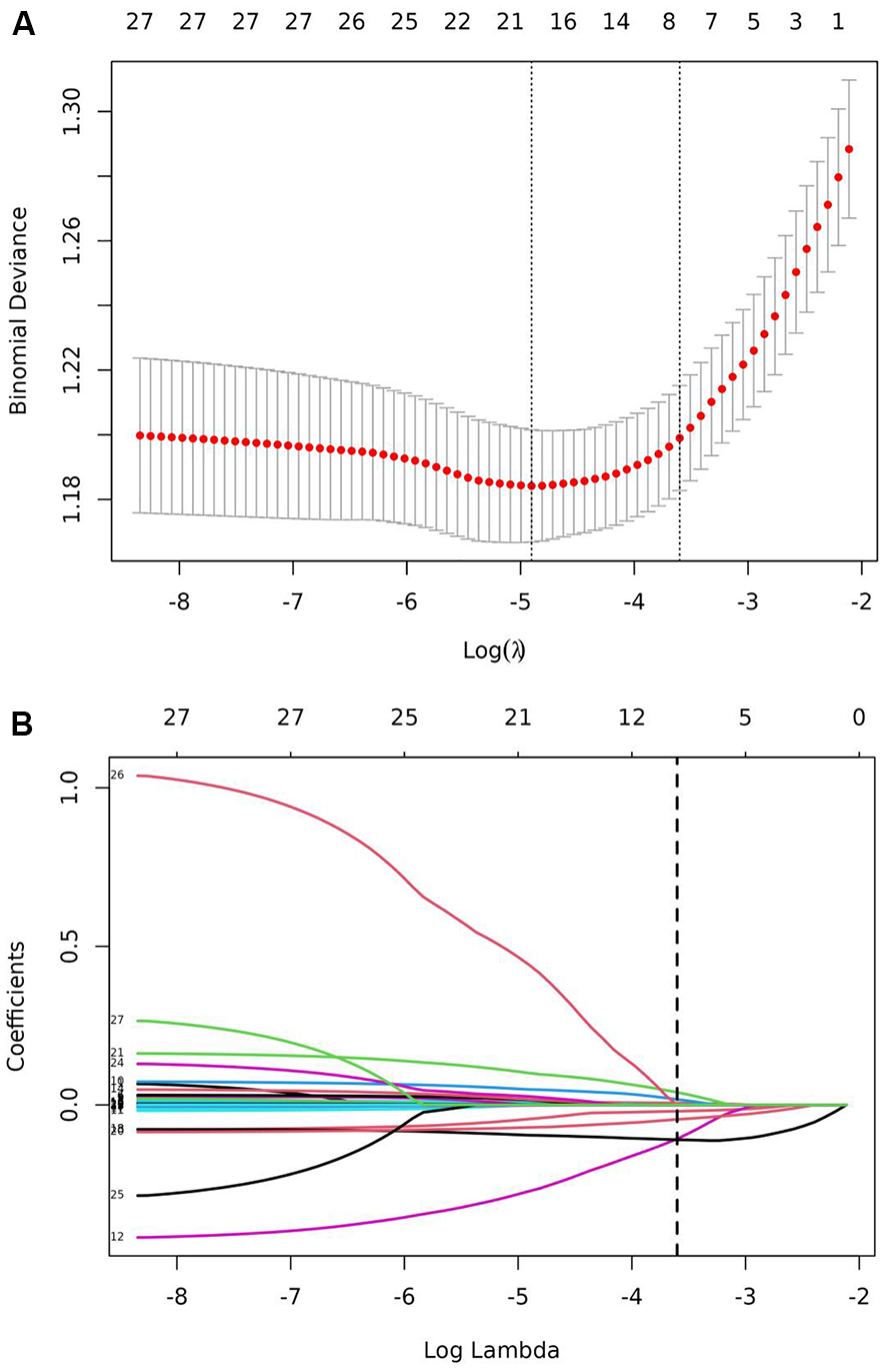
LASSO regression for the screening of the predictor variables. **(A)** LASSO regression cross-validation plot. **(B)** LASSO regression coefficient path plot.

LASSO regression analysis was performed on the training set to identify the variables associated with DPN. A cross-validated error plot of the LASSO regression model is shown in the **Figure 1**. The most regularized and parsimonious model was developed with a cross-validated error within one standard error of the minimum. After 10-fold cross-validation, lambda.1se parameter was selected as 0.027287. At this point, a total of 7 significant variables were finally screened: age (OR = 1.02, 95%CI: 1.01 - 1.03), HC (OR = 0.94, 95%CI: 0.92 – 0.97), FPG (OR = 1.06, 95%CI: 1.01 - 1.11), FCP (OR = 0.66, 95%CI: 0.56 - 0.77), PCP (OR = 0.78, 95%CI: 0.72 – 0.84), ALB (OR = 0.90, 95%CI: 0.87 – 0.94) and BUN (OR = 1.08, 95%CI: 1.01 - 1.17).

### 3.3 Construction of the nomogram

**Figure 2:**
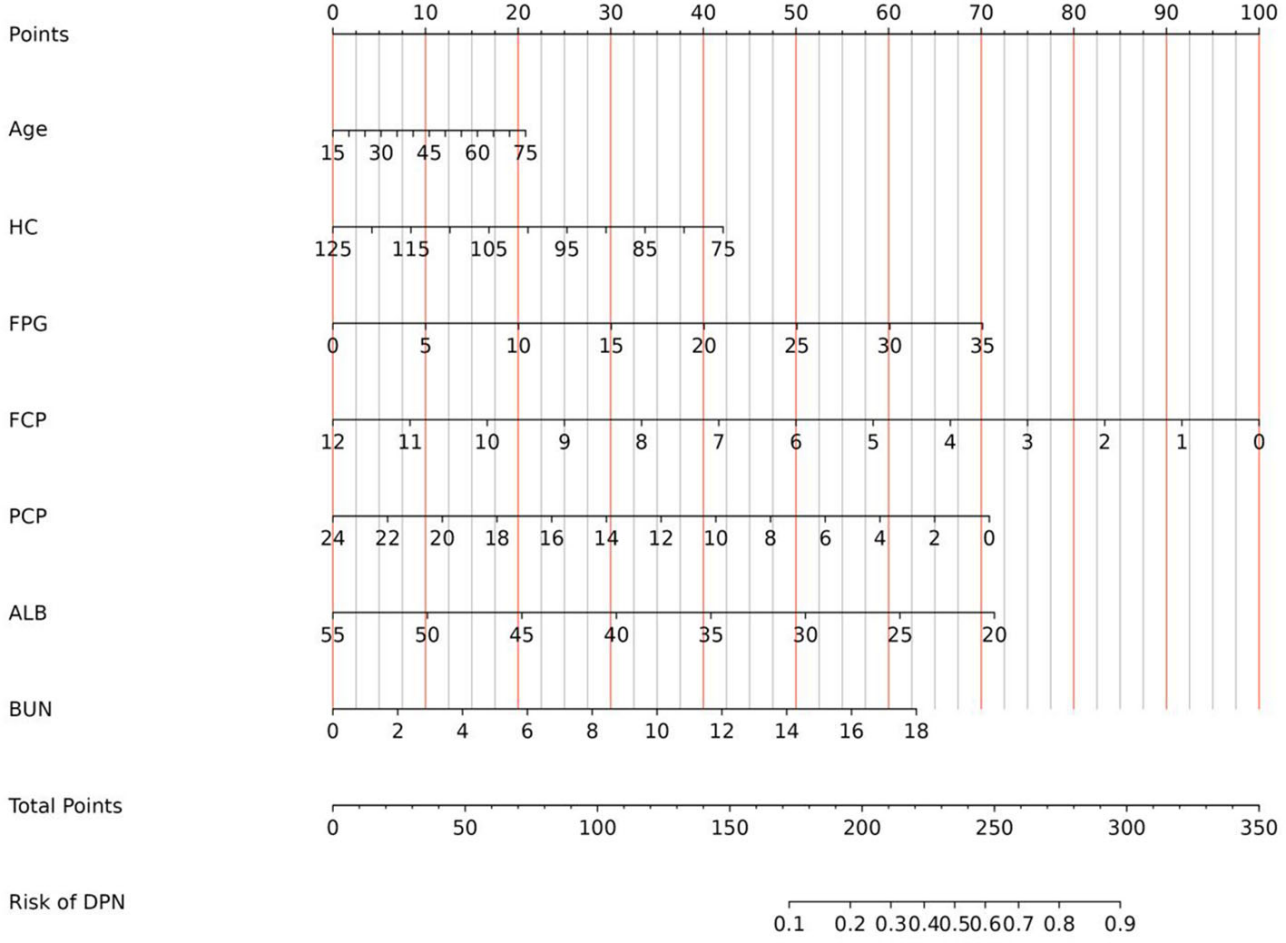
Nomogram for predicting the risk of DPN in diabetic patients. DPN, diabetic peripheral neuropathy; HC, hip circumference; FPG, fasting plasma glucose; FCP, fasting C-peptide; PCP, 2 hour postprandial C-peptide; ALB, albumin; BUN, blood urea nitrogen.

Independent risk factors screened by LASSO regression analysis in the training set were subsequently entered into the prediction model. Consequently, a nomogram model incorporated 7 significant risk factors for predicting DPN was constructed (**Figure 2**). As displayed in the figure, the corresponding parameters from top to bottom were inclusive of the individual points, age, HC, FPG, FCP, PCP, ALB, BUN, the total points, and the risk of developing DPN. Each parameter was assigned a score on the point scale axis. The individual scores of all parameters were aggregated to determine the total points, which can be employed to estimate the probability of developing DPN.

### 3.4 Validation and performance of the nomogram

**Figure 3:**
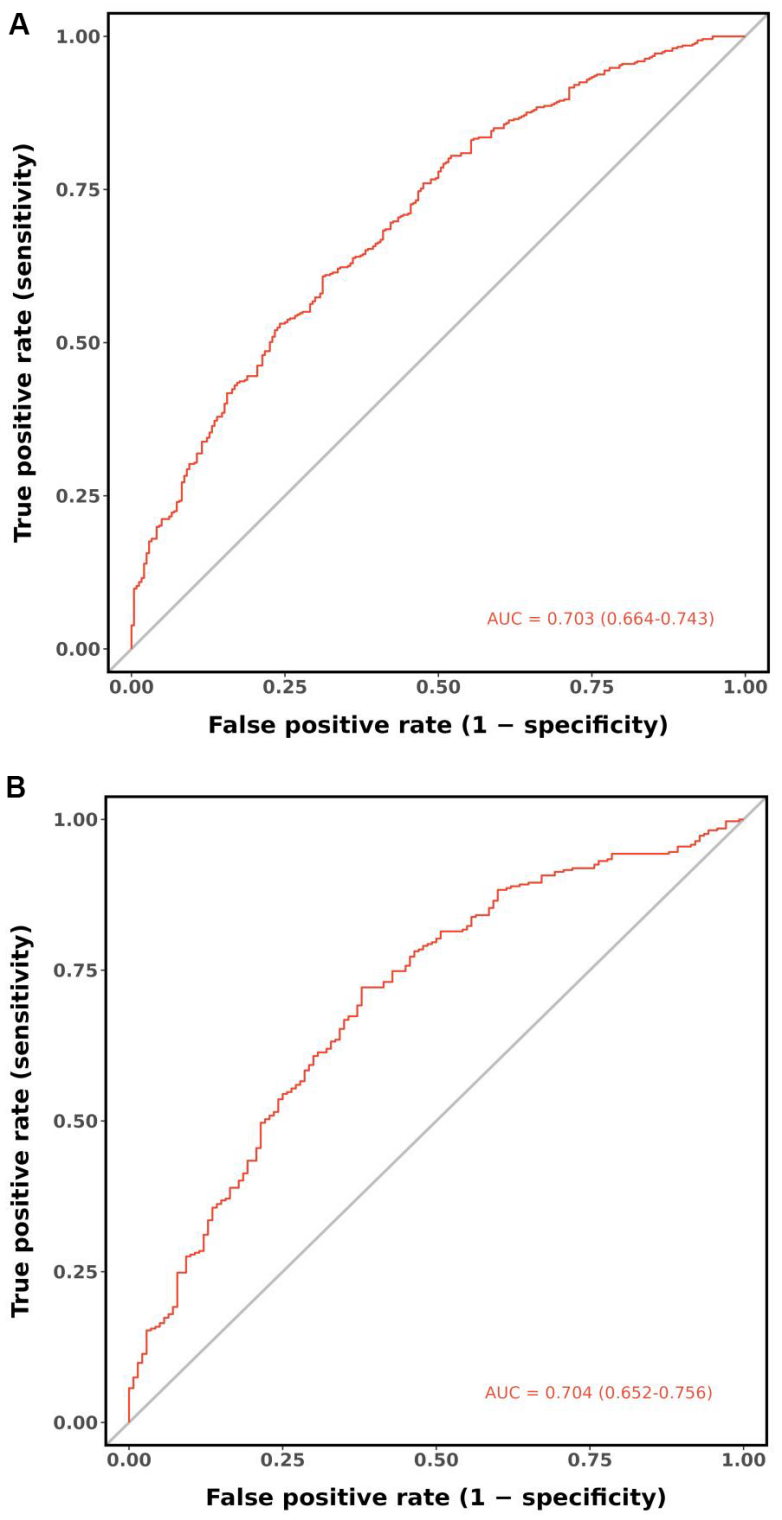
ROC curves of the nomogram. **(A)** Training set. **(B)** Validation set. ROC, receiver operating characteristic; AUC, area under the ROC curve.

**Figure 4:**
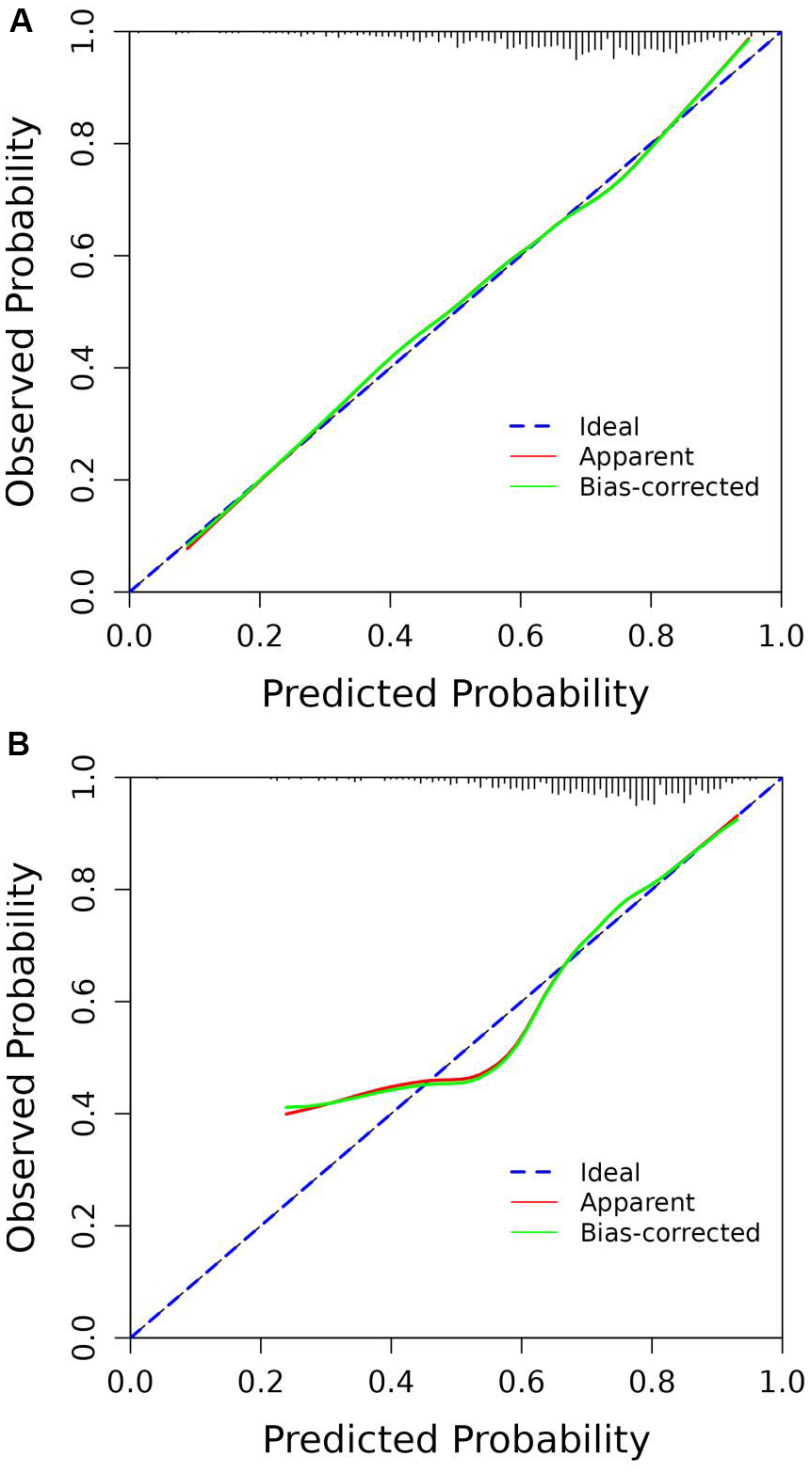
Calibration curves of the nomogram. **(A)** Training set. **(B)** Validation set.

**Figure 5:**
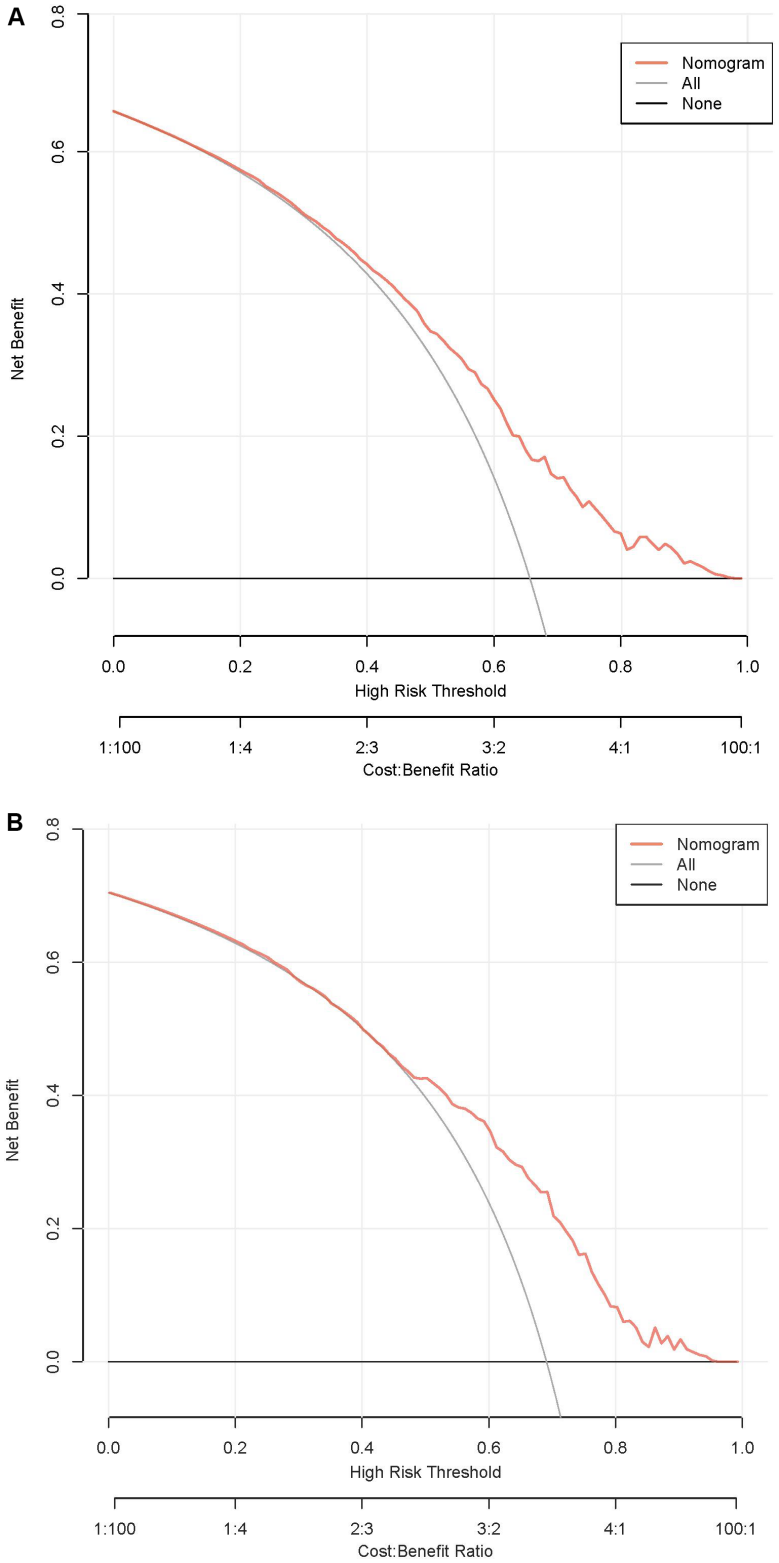
DCA curves of the nomogram. **(A)** Training set. **(B)** Validation set. DCA, decision curve analysis.

According to the ROC analysis, the AUC of the nomogram for the training set was 0.703 (95% CI 0.664-0.743), with a sensitivity of 68.9% and a specificity of 60.8%; the AUC of the nomogram for the validation set was 0.704 (95% CI 0.652-0.756) with a sensitivity of 67.1% and a specificity of 62.0%, indicating the relatively good accuracy and discriminatory power of the model (**Figure 3**). The positive predictive value was 0.479 (95% CI 0.426 - 0.531) in the training set and 0.425 (95% CI 0.360 - 0.491) in the validation set; and the negative predictive value was 0.789 (95% CI 0.747 - 0.831) in the training set and 0.818 (95% CI 0.771 - 0.866) in the validation set. The p-values of Hosmer–Lemeshow test were 0.943 and 0.381 in the training and validation sets respectively, emphasizing an appreciable consistency between the predicted and actual probabilities (p>0.05). To prevent overfitting of the model, internal validation was performed on the training set by bootstrapping techniques and the average AUC obtained after 200 iterations was 0.704 (95% CI 0.701-0.707). In addition, the calibration curves were obtained through the bootstrapping validation of the model with a total of 1000 repetitions. The ‘apparent’ and the bootstrap-corrected ‘bias-corrected’ line in the calibration curves of the nomogram presented a strong resemblance to the ideal diagonal line, indicating a close alignment between the predicted probabilities and the actual probabilities (**Figure 4**). This observation accentuated the high level of prediction accuracy that the nomogram exhibited in both the training and validation sets. **Figure 5** illustrated the DCA for predicting the possibility of DPN and evaluating the clinical utility of the model. The decision curves demonstrated that with a threshold probability of 0.15-0.97 in the training set and 0.20-0.95 in the validation set, the nomogram yielded greater net benefits as compared to the “full treatment” or “no treatment” strategies. The results suggested that the model serves as an effective evaluation tool holding considerable value in the clinical setting.

## 4 Discussion

DPN is a significant neurological complication of DM and a major contributor to diabetic foot syndrome characterized by painful neuropathic symptoms, paresthesia and sensory deficits leading to increased risk of burns, injuries, infections, foot ulcerations and even amputations (1–6). This reinforces the critical importance of early screening and diagnosis of DPN, which can facilitate effective interventions to halt the progression and reduce the morbidity of these severe complications.

As an acknowledged gold standard for the early subclinical diagnosis of DPN, electrophysiological testing offers an objective and quantitative method for detecting peripheral neuropathy. The sensitivity of nerve conduction studies is superior to the clinical examinations of peripheral neuropathy (2–8). However, the prohibitive cost and inconvenience considerably limit the widespread application in large-scale screening or routine clinical practice. Recognizing these challenges, our study aimed to create a workable solution by developing a nomogram for predicting the risk of DPN. This model is designed to facilitate a faster, more efficient, and early assessment of DPN and advance patient care amidst resource constraints.

The nomogram derived from LASSO regression analysis was ultimately composed of age, HC, FPG, FCP, PCP, ALB and BUN. It exhibited good discriminative power and clinical utility in both the training and validation sets, making it a notably convenient and practical tool for clinical use.

Previous literatures have suggested that advancing age is a notable risk factor for DPN. The prevalence of DPN has been shown to increase with age, reaching a peak in more than half of all patients with type 2 diabetes aged 60 years or older (4). Wang W et al. found that the DPN severity was accentuated in those aged over 60 years (11). The univariate and multivariate analyses from their study further indicated that age groups ≥50 years were 1.512-1.853 times more likely to develop DPN than those < 40 years. Similarly, Cheng Y et al. observed that patients older than 66 years had 2.65 times greater probability of developing DPN compared to those < 66 years old (12). DPN is well-recognized as a chronic complication that takes time to develop. It is hypothesized that the primary pathophysiological mechanisms contributing to the onset of DPN are associated with inflammation, oxidative stress and mitochondrial dysfunction, which typically occur during the aging process (1, 4, 13, 14). As individuals age, chronic inflammation and oxidative stress in nerve cells intensify, while the function of mitochondria in these nerve cells gradually declines. This culminates in an insufficient supply of cellular energy, subsequently triggering cellular apoptosis (14).

Prior epidemiological studies have demonstrated that obesity represents a significant risk factor for developing DPN (15–18). A study was performed to investigate the relationship between body composition and DPN (16). The study demonstrated that the presence of DPN was associated with visceral fat area (OR = 1.026; 95% CI, 1.005–1.048) after adjusting for age, sex, diabetes duration, and smoking status. It was thus concluded that abdominal obesity was associated with DPN. It is well known that abdominal obesity is characterized by the accumulation of visceral fat, with WC commonly used in clinical practice as an approximate measure of visceral fat content (19). In contrast, subcutaneous fat accumulates predominantly in the gluteal and lower extremity regions, making HC a widely employed indicator for the assessment of subcutaneous adipose tissue. The findings of our study indicated that HC was negatively associated with DPN, thereby acting as a protective factor for DPN. Similarly, Zhen Q et al. found that leg subcutaneous fat played a protective role in the development of DPN. The risk of developing DPN was reduced by 30% for every 1kg increase in subcutaneous leg fat (17). Appropriate subcutaneous fat mass may exhibit neuroprotective properties due to the lower lipolytic activity of subcutaneous adipose tissue and the preferential absorption of plasma free fatty acids, thereby protecting skeletal muscle from high levels of free fatty acids (17, 20).

It has been proposed by several studies that hyperglycemia and impaired pancreatic islet function are the initiating factors for a range of pathophysiological changes in DPN (21–25). The deterioration of nerve fibers resulting from hyperglycemia gives rise to an imbalance between the nerves responsible for damage and those engaged in repair (23). Abnormal glucose precipitates alterations in mitochondrial function, inflammation, oxidative stress, specific gene transcription and expression, ultimately resulting in neuronal-glial cell damage (24). Additionally, increasing evidence suggests that C-peptide (CP) exerts a range of effects on metabolism, neuroprotection and anti-apoptosis (25–27), including the Na+/K+- ATPase activity, endothelial nitric oxide synthase, expression of neurotrophic factors, and the regulation of molecular species underlying the degeneration of the nodal apparatus in nerves of T1DM (25, 26). A community-based study in China revealed a negative correlation between FCP, PCP levels and DPN in T2DM after adjusting for potential confounders (27). They further proposed that residual β-cell function, as reflected by CP concentrations, exhibits a significant neuroprotective role. This highlights the necessity of minimizing drug-induced β-cells overstimulation and initiating insulin therapy at the optimal juncture to maintain endogenous β-cell activity during the T2DM progression (27).

The relationship between ALB and DPN has been investigated extensively in various studies. Specifically, a study conducted by Yan P et al. demonstrated a negative correlation between serum ALB levels and increased risk of DPN (28). In their study, ALB remained significantly associated with increased risk of DPN after adjusting for demographic, metabolic, inflammation and oxidative stress, and other parameters (OR = 0.499, 95% CI 0.385–0.645, P < 0.01). Furthermore, the optimal cutoff point of ALB linked with the prevalence of DPN was determined to be 39.95 g/L according to ROC analysis (28). In agreement with previous reports, our study also confirmed that ALB was negatively associated with DPN. Synthesized and secreted by hepatocytes, ALB is a main protein component of blood plasma in human body. As an indicator of the body’s nutritional state, ALB is potentially neuroprotective and exhibits a range of physiological features including antioxidant, anti-inflammatory, and anti-thrombotic activities (28, 29). Moreover, it averts apoptosis in endothelial cells, Schwann cells and neurons. It also shields neurons from injuries caused by ischemia and reperfusion, and enhances neurological functional recovery (28–31). Thus, elevated capillary permeability to ALB in diabetic patients can induce basement membrane thickening, interstitial ALB retention and secondarily neurogenic edema. This series of events could result in severe neurological lesions and foster the progression of DPN (32, 33).

A large number of evidences lend support to the observed positive correlation between BUN and the risk of DPN (34–36). Urea nitrogen, a crucial component of urea, mainly emerges as a byproduct of protein metabolism within the body. This substance, excreted through glomerular filtration, is a key metric in assessing renal function, thereby revealing the risk of indirectly related diseases (35, 36). Increased levels of BUN possess the potential to directly affect the function of pancreatic β-cell. Such interference can disturb glucose homeostasis and eventually result in the development of DM and impairment of renal function (37). Along with diabetic nephropathy, DPN is recognized as one of the microvascular complications of DM. There exists a significant link between both conditions at different stages in patients with type 2 diabetes (38). The prevalent understanding posits that the evolution and progression of diabetic nephropathy and DPN occur concurrently due to the exposure of peripheral nerves and renal vessels to the deleterious elements present in diabetic environment (35). There is an imperative necessity for more robust and scientifically rigorous studies to investigate the links between BUN and DPN, thereby illuminating the processes through which BUN affect the pathogenesis of DPN.

A number of prediction models for DPN have been developed in the reviewed literature. Fitri A et al. developed a scoring system prediction model for the severity of diabetic polyneuropathy based on vitamin D levels (39). This model indicated that patients with scores above 4 had a 2.7-fold higher risk of severe DPN. However, it should be noted that this study included only 50 patients with DM and did not indicate specific differences in the risk of developing DPN when specific scores differed. Another study had also devised a nomogram (40); however, the study population was limited to a community-based cohort of 700 cases. Additionally, the diagnostic criteria of DPN based on scores from the Toronto Clinical Scoring System test may result in false positives.

The principal benefit of this study is that the derived nomogram, based on a sample size of 1185 patients, incorporates both anthropological parameters and conventional serum biochemical indicators that are readily accessible in general healthcare settings. Furthermore, our nomogram is the inaugural predictive model to concentrate on the correlation between C-peptide and DPN, thereby elucidating the influence of islet function. The nomogram exhibits favorable discriminatory and predictable properties, rendering it a valuable tool for clinical practice.

There are several certain limitations in our study. Firstly, it was a single-center study with an inevitable degree of internal bias and a lack of generalization to diverse populations. Secondly, as a retrospective cross-sectional study, it was unable to accurately ascertain the precise sequence and causality between the exposure and outcome variables.

Further external validation of the nomogram model in various regions and ethnic groups is required to mitigate selection bias and confirm the generalizability and applicability of the model in different clinical settings. In addition, prospectively designed studies containing more novel indicators or biomarkers are necessary to enhance the predictive accuracy of the nomogram and provide a more favorable and reliable reference for clinical assessment of DPN risk.

## 5 Conclusion

In conclusion, the nomogram prediction model developed in our study consists of age, HC, FPG, FCP, PCP, ALB and BUN. It exhibits good predictive ability and clinical benefits, thus endowing clinicians with an intuitive and practical tool for early identification of individuals with a high risk of DPN in a wide range of clinical settings, especially in centers with limited medical resources.

## Data Availability

The raw data supporting the conclusions of this article will be made available by the authors, without undue reservation.
